# A 21-Year Survey of Escherichia coli from Bloodstream Infections (BSI) in a Tertiary Hospital Reveals How Community-Hospital Dynamics of B2 Phylogroup Clones Influence Local BSI Rates

**DOI:** 10.1128/msphere.00868-21

**Published:** 2021-12-22

**Authors:** Irene Rodríguez, Ana Sofia Figueiredo, Melissa Sousa, Sonia Aracil-Gisbert, Miguel D. Fernández-de-Bobadilla, Val F. Lanza, Concepción Rodríguez, Javier Zamora, Elena Loza, Patricia Mingo, Claire J. Brooks, Rafael Cantón, Fernando Baquero, Teresa M. Coque

**Affiliations:** a Department of Microbiology, Ramón y Cajal University Hospital and Ramón y Cajal Health Research Institute (IRYCIS), Madrid, Spain; b Department of Biology, University of Aveiro, Aveiro, Portugal; c Bioinformatics Unit, Ramón y Cajal University Hospital and Ramón y Cajal Health Research Institute (IRYCIS), Madrid, Spain; d Biomedical Research Networking Center on Infectious Diseases (CIBERINFEC), Madrid, Spain; e Biomedical Research Networking Center for Epidemiology and Public Health (CIBERESP), Madrid, Spain; f Biostatistics Unit, Ramón y Cajal University Hospital and Ramón y Cajal Health Research Institute (IRYCIS), Madrid, Spain; JMI Laboratories

**Keywords:** *Escherichia coli*, ExPEC, bacteremia, ST131, ST73, ST95, ST12, ST127, pandemic clones, long-term clonal dynamics, clonal dynamics

## Abstract

This is a longitudinal study comprising 649 Escherichia coli isolates representing all 7,165 E. coli bloodstream infection (BSI) episodes recorded in a hospital (1996 to 2016). Strain analysis included clonal identification (phylogenetic groups/subgroups, STc131 subclades, pulsed-field gel electrophoresis [PFGE], and whole-genome sequencing [WGS]), antibiotic susceptibility (13 antibiotics), and virulence-associated genes (VAGs; 29 genes). The incidence of E. coli BSI increased from 1996 to 2016 (5.5 to 10.8 BSI episodes/1,000 hospitalizations, average 7 to 8/1,000). B2 isolates predominate (53%), with subgroups B2-I (STc131), B2-II, B2-IX, and B2-VI representing 25%, 25%, 14%, and 9%, respectively. Intertwined waves of community-acquired (CA) plus health care-associated and community-onset health care-associated (HCA) and hospital-acquired (HA) episodes of both B2 and non-B2 phylogroups occurred. A remarkable increase was observed only for B2-I-STc131 (C1/C2 subclades), with oscillations for other B2 subgroups and phylogroups throughout the years. Epidemic and persistent clones (comprising isolates with highly similar/identical PFGE types and genomes differing in 6 to 173 single nucleotide polymorphisms [SNPs]) of B2-I (STc131), B2-II (STc73), B2-III (STc127), B2-IX (STc95), and B2-VI (STc12) were recovered from different patients, most at hospital admission, for long periods (2 to 17 years), and extended-spectrum beta-lactamase (ESBL) producers or resistance to ciprofloxacin in B2 isolates was almost restricted to B2-I (STc131) subclade C. STc131 contributed to increasing the B2 rates but only transiently altered the E. coli population structure. The increase of E. coli BSI was determined by waves of CA+HCA BSI episodes that predate the waves of HA BSI. Besides the risk of hospital transmission that led to temporal increases in BSI, this study suggests that E. coli populations/clones from community-based healthy individuals may occasionally have an epidemic structure and provide a source of transmissible strains influencing the HA BSI incidence.

**IMPORTANCE** Sepsis is the third leading cause of mortality in Western countries and one of the Global Health Threats recognized by the WHO since 2017. Despite Escherichia coli constituting the most common cause of bloodstream infections (BSI), its epidemiology is not fully understood, in part due to the scarcity of local and longitudinal studies. Our work analyzes the long-term dynamics of E. coli causing bacteremia in a single institution and reveals waves of different clonal lineages that emerge periodically and successfully spread afterward in both the community and hospitals. Because the origin of E. coli bloodstream infections is the gut, the microbiota of healthy individuals might occasionally have an epidemic structure, providing a source of E. coli strains to influence the incidence of hospital BSI. The study complements previous fractionated observations focusing on specific E. coli lineages or antibiotic-resistant isolates in the last decades and helps to understand the epidemiology of E. coli BSI and the dynamics of pandemic clones.

## INTRODUCTION

The increasing and progressive annual rate of bloodstream infection (BSI) episodes at a global level (9 to 13% in Western countries) led the WHO to consider sepsis a Global Health Threat in 2017 (https://www.global-sepsis-alliance.org/news/2017/5/26/wha-adopts-resolution-on-sepsis). The problem affects more than 30 million people in the world and represents the third leading cause of mortality in Europe and North America (1–4). Escherichia coli, a commensal opportunistic pathogen of humans and animals, constitutes the primary cause of BSI (5, 6).

The gut microbiota is often the origin of all extraintestinal infections caused by E. coli (7) and varies between humans of different ages and lifestyles (7, 8). Among the 7 major phylogenetic groups of the species (A, B1, B2, C, D, E, and F), B2 is nowadays predominant in clinical and fecal isolates from adults and children of Western areas (7–9). Despite the apparent persistent structure of E. coli in the gut of these individuals, clonal expansions of emerging sequence type complexes (STcs) have periodically occurred (10, 11). Currently, several E. coli phylogenetic groups and some of the 10 B2 E. coli subgroups known (B2-I to B2-X) are overrepresented by certain sequence type complexes (STcs) (e.g., B2-I [STc131], B2-II [STc73], B2-IX [STc95], D [STc69]. or F [STc648]), although their abundance and diversity vary between human populations (10, 12–14).

E. coli bacteremia was not considered common at the beginning of the 20th century, but it has steadily increased for decades according to long surveys performed at Boston City Hospital (Boston, MA) between 1935 and 1972 (15), at St. Thomas Hospital in London, England, between 1969 and 1986 (16), and, more recently, at hospitals in Western countries (most are cross-sectional or longitudinal multicentric studies) (1, 4, 17–19). The survey performed during the 1980s reflected, for the very first time, an incidence peak of BSI caused by E. coli coincident with a community-based clonal outbreak caused by E. coli O15:H12 which led to its introduction in the hospital setting and a subsequent increase in nosocomial BSI cases (16). Shortly afterward, community-based and hospital clonal outbreaks by E. coli of phylogroup D were extensively reported during the 1990s, namely, ST69 in the United States and ST393 O15:H12 in the United Kingdom and Spain (10). The most comprehensive recent analysis, using European Antimicrobial Resistance Surveillance System (EARSS) data from 2002 to 2008, also highlighted a remarkable average annual increase of 29.9% in the number of reported bacteremia cases caused by E. coli isolates resistant to third-generation cephalosporins (3GCs) (1), although phylogenomic data were not provided in that publication. Nonetheless, many studies performed during the 2000s and afterward documented the increase of community-acquired (CA) outbreaks of B2 E. coli lineages such as STc73 (B2-II), STc95 (B2-IX), and, more recently, STc131 (B2-I). Currently, all them are considered “pandemic clones” due to their global predominance (13, 17). The current knowledge suggests differences between countries, but such information is highly fractionated in multicentric studies (1), is mostly focused on antibiotic-resistant BSI isolates (20), and is performed at variable periods of time using different sampling criteria (1–4, 19, 20). Only studies from the United Kingdom, where surveillance of BSI has been compulsory since 2011, provide a long-term comprehensive analysis of BSI and the population structure and dynamics of E. coli causing BSI (4, 17).

Unfortunately, clonal expansions at the local level have been analyzed poorly and mostly from the perspective of antimicrobial resistance and for very limited periods of time. However, local settings are important sources of information because they reflect the dimensions of human population structure (age, sex, interconnectedness) and offer stability in terms of the intensity of selection (e.g., common policies to control antimicrobial resistance such as antibiotic use and infection control strategies), models of health care delivery, and measuring approaches (diagnostic tools) (1, 19), all these issues being important to the epidemiology of infectious agents (21, 53–55).

We retrospectively studied a randomized sample of 649 isolates drawn from a collection of 7,165 E. coli isolates, which represented all BSI episodes registered at our institution between 1996 and 2016. This period coincided with the global emergence and amplification of various *bla*_ESBL_ genes and B2 E. coli clones and with the overall increase in the frequency of E. coli BSI. The aim of this study was to infer the local diversity and dynamics of E. coli strains causing BSI, focusing on the B2 phylogroup.

## RESULTS

### Epidemiology of the 7,165 E. coli isolates causing BSI at Hospital Ramón y Cajal.

**FIG 1:**
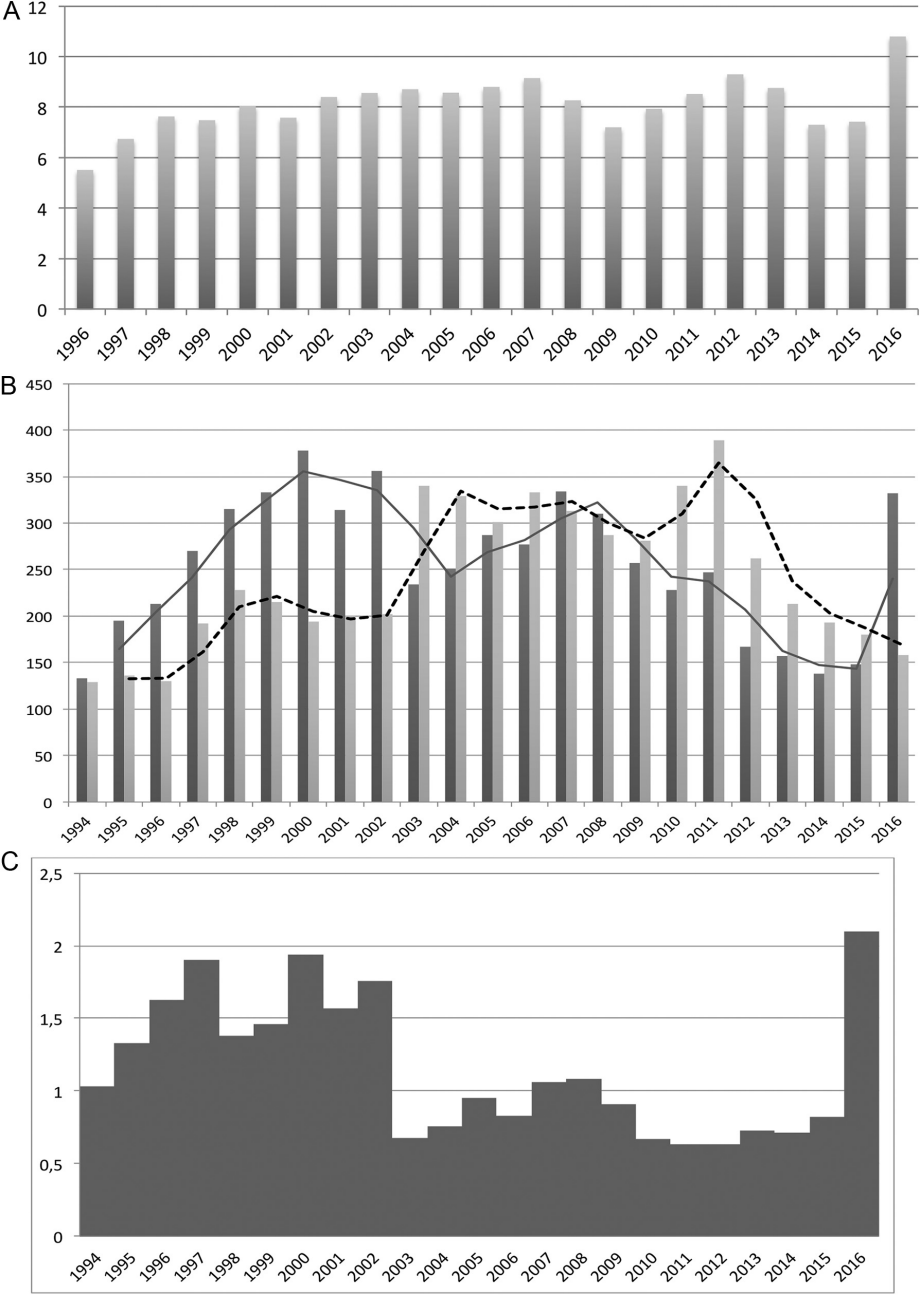
Incidence and origin of BSI episodes caused by Escherichia coli at the Hospital Universitario Ramón y Cajal (1994 to 2016). (A) Incidence of E. coli BSI (episodes/1,000 hospitalizations); (B) occurrence of HCA+CA and HA episodes of BSI; (C) ratio of HCA+CA to HA BSI episodes. Abbreviations: HA, hospital acquired; CA, community acquired; HCA, community-onset health care associated. Bars in black and bars in gray represent HA episodes and CA episodes, respectively. Solid and dashed lines represent the dynamics of HA episodes and CA episodes, respectively.

The incidence of BSI caused by E. coli from 1996 to 2016 in our institution ranges from 5.5 to 10.8 BSI episodes/1,000 hospitalizations, with fluctuations of 7 to 8 BSI episodes/1,000 admissions in most of the years studied. The highest incidence peak, observed in 2016, parallels the blooming of clinical isolates producing CTX-M-27 in our and other hospitals (22). Although the overall numbers of BSI episodes in the hospital and community settings were similar at the beginning of the study in the mid-1990s, we observed a steady increase in both hospital-acquired (HA) BSI and community-acquired (CA) plus health care-associated (HCA) BSI from 1995 to 2002 (CA+HCA/HA ratio >1 to 2) followed by waves of alternative predominance of either CA+HCA BSI or HA BSI. The increases of BSI episodes in the community seem to predate those in the hospital setting and would explain the wave dynamics between the hospital and the community-based populations suggested in the literature and the overall shift in the ratio of BSI acquired in the community and the hospital (Fig. 1). The analysis of antimicrobial resistance records in our department for the blood E. coli isolates revealed a coincidental increase in the rise of BSI and the increasing trends of E. coli resistance to fluoroquinolones (FQ^R^), mainly ciprofloxacin (Cip^R^), from 1994, and resistance to third-generation cephalosporins (3GCs) from 2003. Rates of resistance to other antibiotics remained stable during the period of study (data not shown).

Urinary tract infections (UTIs) were identified as the origin of the BSI in one-third of the cases. This finding was more frequent in women than in men (42.33% versus 26.77%, *P* ≤ 0.005) but not significantly different between patients of different age groups (*P* > 0.05). The proportion of polymicrobial BSI was 9% (*n* = 57/649), and the proportions were similar for men and women (11.8% versus 7.3% of the BSI cases, respectively).

### Clonal diversity of E. coli causing BSI.

The predominant phylogenetic group was B2 (348/649; 53.06%), followed at a much lower frequency by D (11.4%), B1 (7.24%), A (6.3%), C (5.1%), F (6.8%), E (0.9%), and clades I and II (0.9%). Half of the B2 isolates corresponded to subgroups B2-I (25.6%) and B2-II (25.1%), which were followed by B2-IX (14.2%), B2-VI (9.5%), and others (see Fig. S1 in the supplemental material). The STc131 isolates, clearly predominant within the B2-I subgroup, represent 12.3% of the total BSI E. coli isolates and 21.8% of the B2 phylogroup. The STc131 isolates (serogroups O25b and O16 representing 92% and 8%, respectively) split into clade A (7/82, 9%), clade B (16/82, 20%), and clade C (59/82, 71%). Clade C comprised isolates of subclades C1 (29/82, 35.4%), C2 (22/82, 26.8%), C0 (6/82, 7.3%), and C1-M27 (2/82, 2.4%).

10.1128/msphere.00868-21.2 FIG S1 Distribution of E. coli phylogroups and B2 phylogenetic subgroups. (A) E. coli phylogroups; (B) E. coli B2 phylogenetic subgroups; (C) E. coli B2-I (STc131) clades A, B, and C and subclades C0/H30 (FQ^S^), C1/H30-R (FQ^R^), C2/H30-Rx (FQ^R^ + *bla*_CTX-M-15_), and C1-M27/H30-R (FQ^R^ + *bla*_CTX-M-27_). Download FIG S1, PDF file, 0.09 MB. Copyright © 2021 Rodríguez et al. 2021 Rodríguez et al. https://creativecommons.org/licenses/by/4.0/ This content is distributed under the terms of the Creative Commons Attribution 4.0 International license.

### Epidemiological characteristics of B2 E. coli isolates. (i) Acquisition of the BSI.

**FIG 2:**
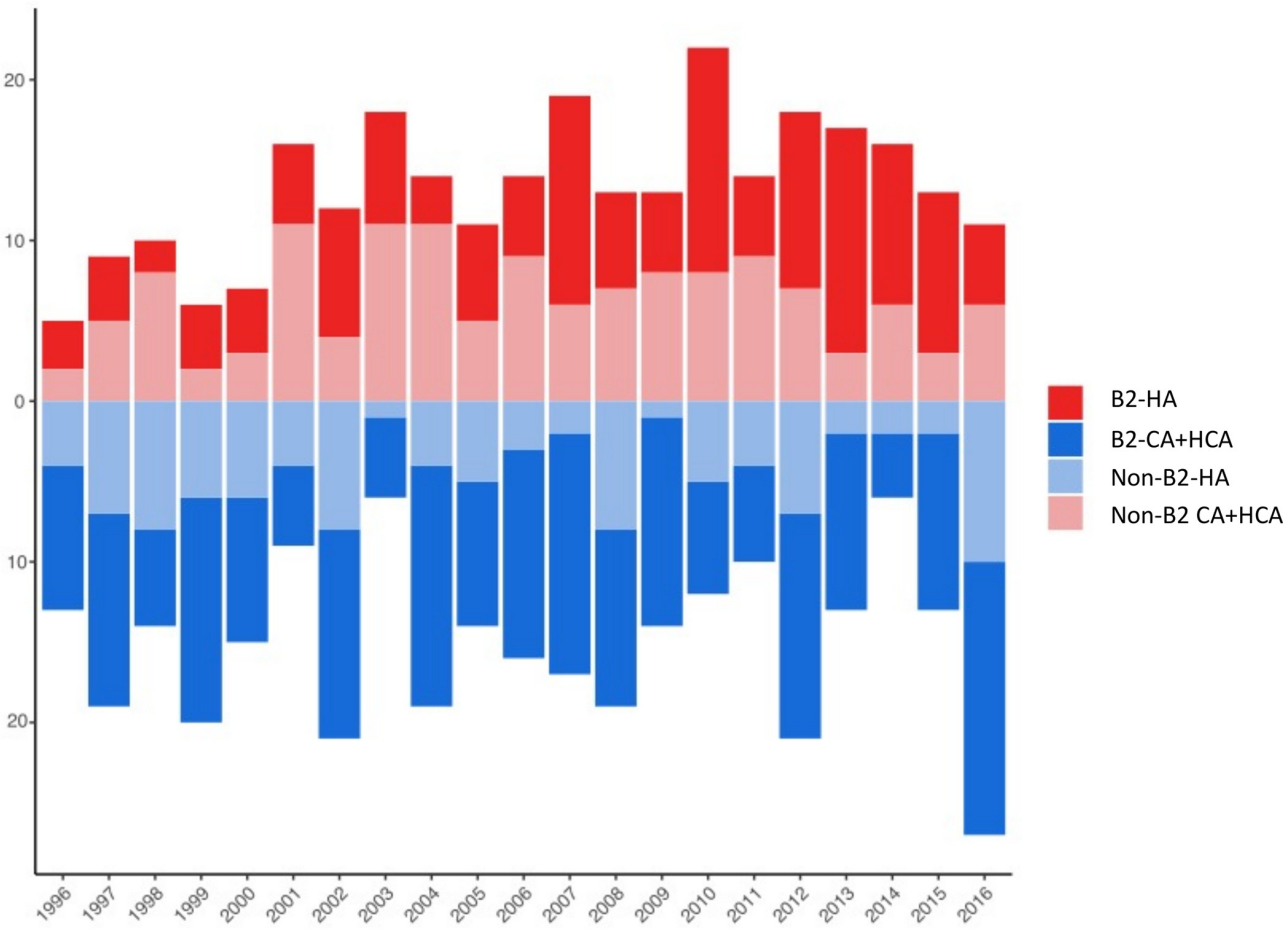
Dynamics of B2 and non-B2 lineages in hospital-acquired (HA) versus community-acquired (CA) plus health care-associated and community-onset health care-associated (CA+HCA) BSI E. coli isolates. Stacked bar plot representing the rates of B2 and non-B2 HA (red and light red bars, respectively) and B2 and non-B2 CA+HCA (blue and light blue bars, respectively).

**FIG 3:**
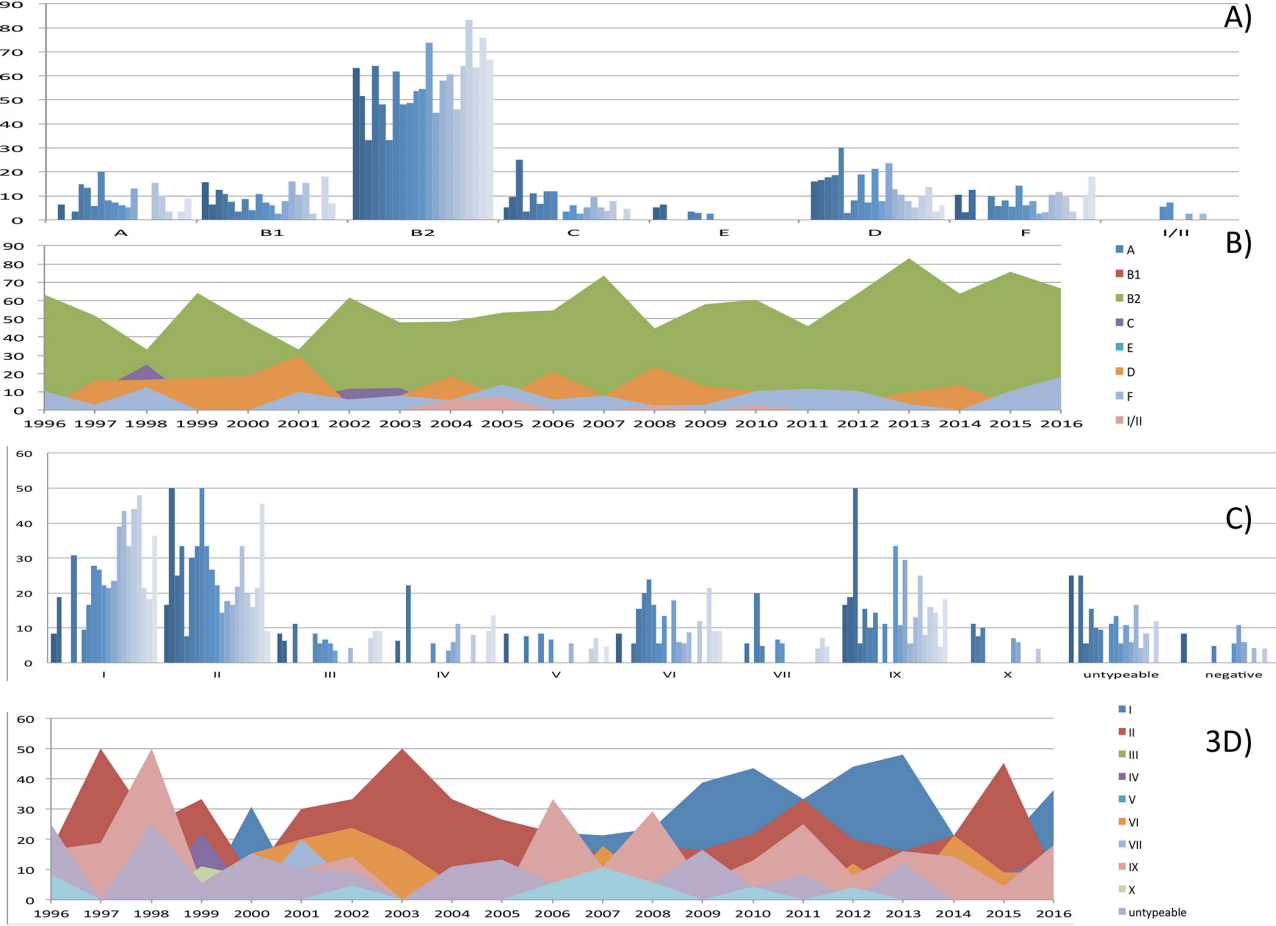
Temporal distribution of E. coli populations at Hospital Universitario Ramón y Cajal (HURyC) (1996 to 2016). (A and B) Temporal distribution of E. coli phylogroups; (C and D) temporal distribution of E. coli B2 subgroups. The *x* axis represents the different phylogroups (panel A) or the different B2 subgroups (panel C). Shading differences reflect the presence of each phylogroup or B2 subgroup per year (1996 to 2016). The *y* axis represents the number of episodes caused by a given phylogroup (panel A and panel B) or by a given B2 subgroup.

Most B2 isolates were recovered from community-based patients although overlapping waves of CA+HCA BSI and HA BSI episodes of strains of both B2 and non-B2 phylogroups occurred until 2008, when B2 apparently overtook non-B2 BSI episodes and STc131 became transiently predominant (Fig. 2 and 3). Oscillations were observed for both CA+HCA and HA episodes although an increasing trend was detected only for the HA ones (Fig. 2). The stratification of the data (CA versus HCA+HA, B2 versus non-B2) made the sample size of each subgroup too small to infer significance. Nonetheless, the data are in agreement with the numbers obtained for all BSI cases (Fig. 1).

Almost half of patients with STc131 (46%) and all with predominant B2-II (STc73), B2-III (STc127), B2-VI (STc12), and B2-IX (STc95) persistent clones were admitted to the medical emergency ward with an established BSI, suggesting the frequent presence of these clones in the community. We also detected the same pulsed-field gel electrophoresis (PFGE) patterns in isolates from different patients for E. coli of B2-I ST131 (clades H22, C1, and C2), B2-II (ST73), B2-III (ST127), B2-IV, B2-VI (ST12), and B2-IX (ST95) (Table S1).

10.1128/msphere.00868-21.9 TABLE S1 Relationship between predominant clonal lineages isolated in HURyC (1996 to 2016). The table reflects the sequence types (STs), PFGE profiles, date of isolation, and hospital ward where the blood culture was taken. Download Table S1, DOC file, 0.06 MB. Copyright © 2021 Rodríguez et al. 2021 Rodríguez et al. https://creativecommons.org/licenses/by/4.0/ This content is distributed under the terms of the Creative Commons Attribution 4.0 International license.

### (ii) Temporal variation.

Except for phylogroups B2-I (ST131) and phylogroup D, the trends of the phylogroups did not significantly change during the period analyzed (Fig. 3). However, the occurrence of major subgroups (B2-II, B2-IX, B2-VI, B-IV, and B2-VII) greatly varied through the years, suggesting episodes of transmission with transient amplifications.

**FIG 4:**
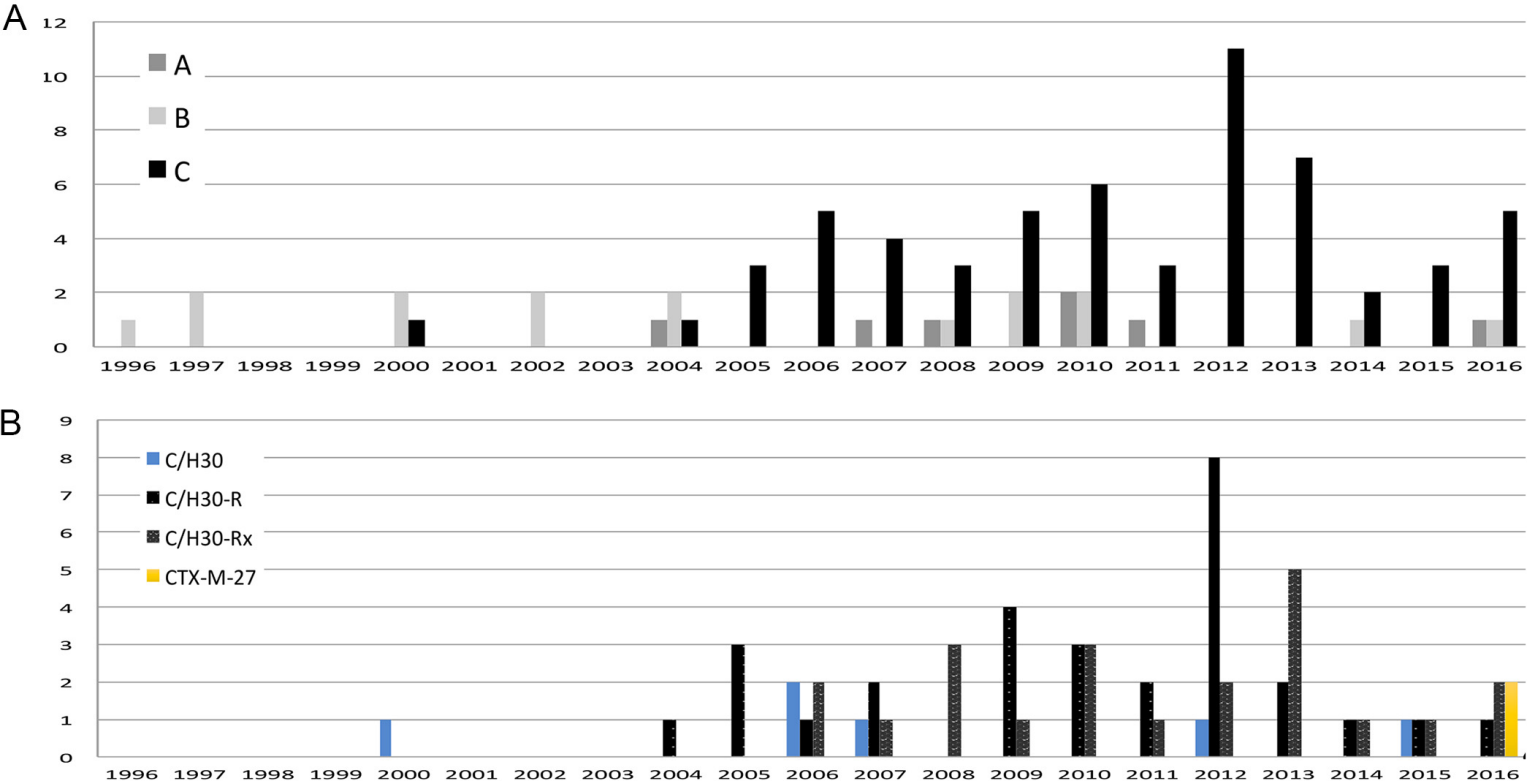
Temporal distribution of E. coli populations at HURyC (1996 to 2016). (A) Distribution of STc131 clades; (B) timeline of the STc131 subclades C0 and C1. The *y* axis represents the number of episodes caused by each ST131 clade (panel A) or subclade (panel B).

The STc131 E. coli was the only group increasingly recovered coinciding with its global amplification (23). The ST131 clade B was initially detected in 1996 and remained steadily identified since then. In the current study, the ST131 of clade A was first detected in 2004, but we had identified STc131 clade A in clinical isolates of TEM-4 and TEM-24 producers from 1991 and 2000, respectively, in other studies by the group (24, 25) For the predominant ST131 clade C, the subclade C0 (H30, FQ^S^) was detected in 2000, followed by C1 (H30-R, FQ^R^) in 2004, C2 (H30-Rx, FQ^R^ *bla*_CTX-M-15_) in 2006, and C1-M27 (H30-Rx, FQ^R^ *bla*_CTX-M-27_) in 2016 (Fig. 4). Further analysis of PFGE and whole-genome sequencing (WGS) revealed clonal amplifications (see below).

### (iii) Distribution by age.

All phylogenetic groups were present in patients of all age groups, although differences were observed for subgroups B2-I (STc131), B2-II, B2-VI, and B2-IX. This distribution implies the clear predominance of B2-I in the elderly (>80 years) and B2-II in individuals younger than 45 years. Others showed a bimodal distribution, such as B2-IX and B2-VI (Fig. S2).

10.1128/msphere.00868-21.3 FIG S2 Diversity of E. coli per age group. (A) Per phylogroup; (B) per B2 subgroup. Half of the B2 isolates corresponded to B2-II and B2-IX (33% and 21%, respectively) for patients between 15 and 45 years, B2-I and B2-II (23% and 25% to 28%, respectively) for patients between 45 and 80 years, and B2-I and B2-IX (36% and 20%, respectively) for patients of >80 years. Download FIG S2, PDF file, 0.05 MB. Copyright © 2021 Rodríguez et al. 2021 Rodríguez et al. https://creativecommons.org/licenses/by/4.0/ This content is distributed under the terms of the Creative Commons Attribution 4.0 International license.

### Antimicrobial susceptibility.

Figure S3 shows the antimicrobial susceptibility patterns in the sample of 649 E. coli isolates, revealing that Cip^R^ appears in between 35% and 50% of the isolates of each phylogroup. Although the lowest Cip^R^ rate corresponded to the B2 phylogroup (17%), it is of note that most Cip^R^ B2 isolates were STc131 (70% versus 3.5% in non-ST131 B2, with major B2 subgroups II, III, IV, VI, and IX being susceptible). Only 6.1% of the total number of B2 isolates showed a 3GC^R^ phenotype compatible with the production of an extended-spectrum beta-lactamase (ESBL), further identified as CTX-M-15, CTX-M-14, and CTX-M-27, and the phenotype was detected only among STc131 isolates. Resistance to ampicillin (70.4%), streptomycin (39.6%), nalidixic acid (32.8%), and co-trimoxazole (45%–50%) was frequent among the isolates of the various phylogroups. Remarkable differences were observed for amoxicillin-clavulanic acid, kanamycin, gentamicin, tetracycline, and chloramphenicol, mostly due to the phylogroup C isolates, all clonally unrelated (data not shown). Susceptibility to the 13 antimicrobials tested was observed in 17% of the total number of isolates tested, with the frequency of susceptible isolates higher among those of phylogroups A, B1, B2, and F (15% each) than those of phylogroups D (10%), E (1%), and C (0%). Within B2, a pansusceptibility pattern was more frequent for non-STc131 than for STc131 isolates (26.8% versus 3.7%). The B2-STc131 strains showed a multiresistant profile (resistance against ≥4 antimicrobial agents of different families) more frequently than other B2 members (56.6% versus 11.6%; *P* < 0.001).

10.1128/msphere.00868-21.4 FIG S3 Antibiotic susceptibility of B2 Escherichia coli causing BSI. Abbreviations: AMC, amoxicillin-clavulanic acid; AMP, ampicillin; CAZ, ceftazidime; CHL, chloramphenicol; CIP, ciprofloxacin; CTX, cefotaxime; GEN, gentamicin; KAN, kanamycin; MEM, meropenem; NAL, nalidixic acid; STR, streptomycin; TET, tetracycline; TMP-SXT, trimethoprim-sulfamethoxazole; susceptible, susceptible to all 13 antibiotics analyzed. Download FIG S3, PDF file, 0.03 MB. Copyright © 2021 Rodríguez et al. 2021 Rodríguez et al. https://creativecommons.org/licenses/by/4.0/ This content is distributed under the terms of the Creative Commons Attribution 4.0 International license.

### VAG profiling in the B2 phylogroup.

**FIG 5:**
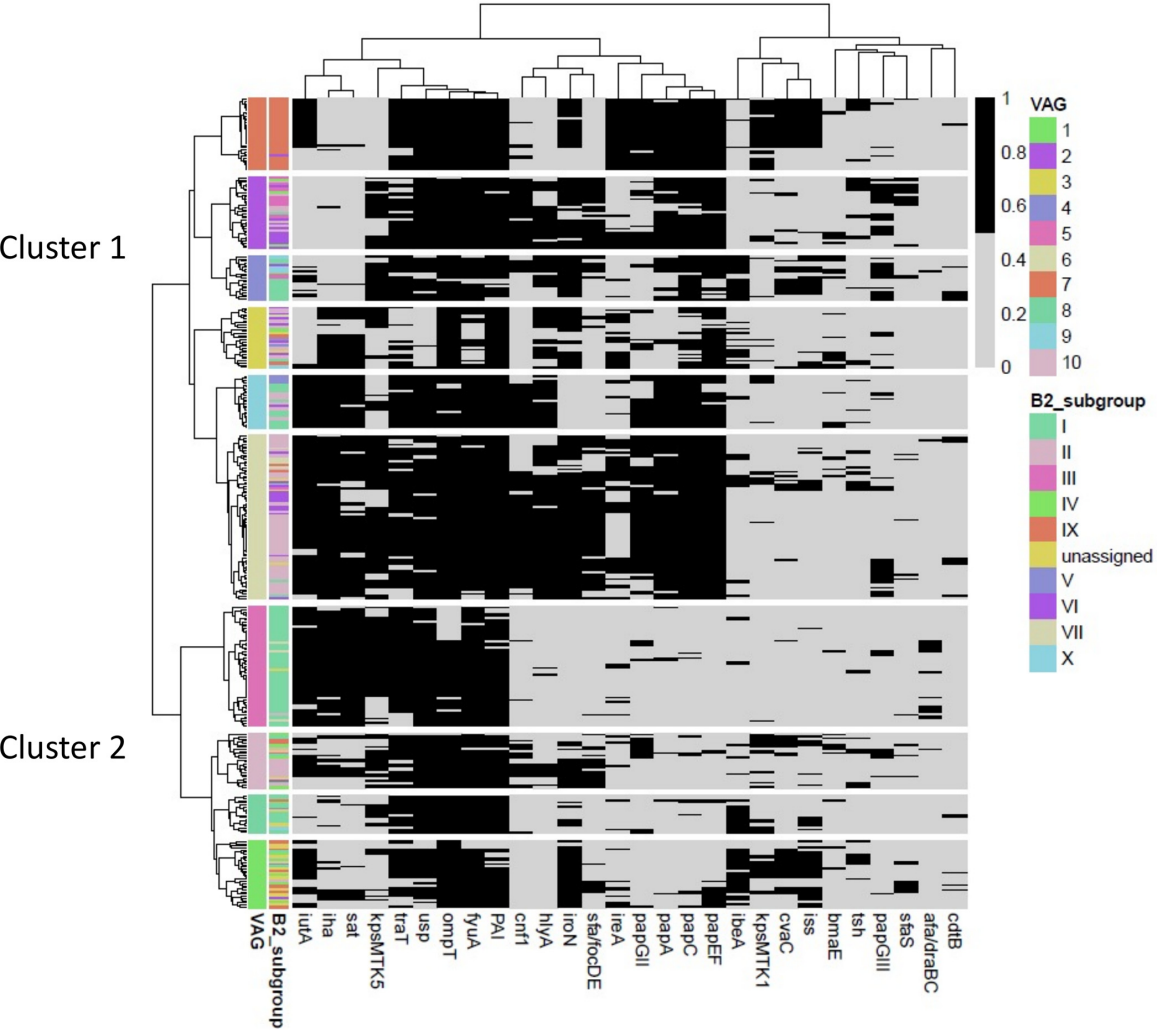
Heatmap of 29 virulence factors (presence/absence) for the 348 B2 phylogenetic group strains causing BSI at HURyC (1996 to 2016). VAGs, virulence-associated genes. VAGs were determined by hierarchical clustering using the Ward method and Jaccard similarity distance. Abbreviations of the VAGs tested: toxins—*hlyA* (α-hemolysin), *sat* (secreted autotransporter toxin, a serine protease), *cnf1* (cytotoxic necrotizing factor 1), *cdtB* (cytolethal distending toxin), and *tsh* (temperature-sensitive hemagglutinin); siderophores—*iroN* (salmochelin receptor), *iutA* (aerobactin synthesis, receptor), *ireA* (iron-regulated element, catecholate siderophore), and *fyuA* (yersiniabactin receptor); adhesins—fimbria type P (*pap*GI, *pap*GII, *pap*GIII, *pap*A, *pap*C, and *pap*EF), *sfa/focDE* (type S fimbriae, *sfa*/*foc* S and F1C fimbriae), *afa/draBC*, adhesin *afa*/*dra* Dr antigen-binding adhesins (AFA I-III, Dr, F1845), *bmaE* (blood group M-specific adhesin), and *iha* (iron-regulated-gene-homologue adhesin); protectins—*kpsMT* II (group II capsule synthesis, e.g., K1, K5, K12), *kpsMT* III group III capsule synthesis (e.g., K3, K10, K54), and *traT* (surface exclusion; serum resistance associated); invasins—*ibe*A-C (invasion of brain endothelium IbeA); miscellaneous—*cvaC* (microcin/colicin V; on plasmids with *traT*, *iss*, and *iuc/iut*), *ompT* (outer membrane protein T), *usp* (uropathogenic-specific protein, bacteriocin), PAI (*malX*, a PAI CFT073 marker), and *iss* (increased serum survival, outer membrane protein).

According to the content of the virulence-associated genes (VAGs), the B2 E. coli strains were clustered into 2 large groups and 10 subgroups, each comprising numerous gene combinations and showing variable redundancies (Fig. 5). While more than half (56%) of the B2-I (STc131) strains clustered in the VAG 1 group, with predominance of the subcluster VAG 1.5, E. coli isolates of B2-II, B2-III, B2-V, B2-VII, B2-IX, and B2-X (54 to 75%) clustered in the VAG 2 group and, more specifically, in the VAG subgroups 2.6, 2.2, 2.9, 2.6, 2.7, and 2.4, respectively (ordered by frequency). Despite some VAG variability within each B2 phylogenetic subgroup, the results suggest a conserved genetic structure related to virulence and colonization in the B2 strains analyzed in this study, in part due to the presence of some persistent (“epidemic”) strains identified through the years. A detailed analysis of virulence content is provided in Text S1 and Fig. S4, S5, and S6.

10.1128/msphere.00868-21.1 TEXT S1 Virulence-associated gene profiling in the B2 phylogroup of Escherichia coli. Download Text S1, DOCX file, 0.06 MB. Copyright © 2021 Rodríguez et al. 2021 Rodríguez et al. https://creativecommons.org/licenses/by/4.0/ This content is distributed under the terms of the Creative Commons Attribution 4.0 International license.

10.1128/msphere.00868-21.5 FIG S4 Heatmap displaying the relative abundance of B2 phylogenetic groups and VAG profiles. Variables showing goodness-of-fit are shown in red, whereas dark blue regions indicate poor frequencies. Clustering of the B2 subgroups and VAGs and analysis of the assembly statistics were performed using a hierarchical clustering method in R (hclust). Abbreviations of the VAGs tested: toxins—*hlyA* (α-hemolysin), *sat* (secreted autotransporter toxin, a serine protease), *cnf1* (cytotoxic necrotizing factor 1), *cdtB* (cytolethal distending toxin), and *tsh* (temperature-sensitive hemagglutinin); siderophores—*iroN* (salmochelin receptor), *iutA* (aerobactin synthesis, receptor), *ireA* (iron-regulated element, catecholate siderophore), and *fyuA* (yersiniabactin receptor); adhesins—fimbria type P (*pap*GI, *pap*GII, *pap*GIII, *pap*A, *pap*C, *pap*EF), *sfa/focDE* (type S fimbriae, *sfa*/*foc* S and F1C fimbriae), *afa/draBC*, adhesin *afa*/*dra* Dr antigen-binding adhesins (AFA I-III, Dr, F1845), *bmaE* (blood group M-specific adhesin), and *iha* (iron-regulated-gene-homologue adhesin); protectins—*kpsMT* II (group II capsule synthesis, e.g., K1, K5, K12), *kpsMT* III group III capsule synthesis (e.g., K3, K10, K54), and *traT* (surface exclusion; serum resistance associated); invasins—*ibe*A-C (invasion of brain endothelium IbeA); miscellaneous—*cvaC* (microcin/colicin V; on plasmids with *traT*, *iss*, and *iuc/iut*), *ompT* (outer membrane protein T), *usp* (uropathogenic-specific protein, bacteriocin), PAI (*malX*, a PAI CFT073 marker), and *iss* (increased serum survival, outer membrane protein). Download FIG S4, PDF file, 0.10 MB. Copyright © 2021 Rodríguez et al. 2021 Rodríguez et al. https://creativecommons.org/licenses/by/4.0/ This content is distributed under the terms of the Creative Commons Attribution 4.0 International license.

10.1128/msphere.00868-21.6 FIG S5 Heatmap displaying the relative abundance of B2 phylogenetic groups and VAGs. Variables showing goodness-of-fit are shown in red, whereas dark blue regions indicate poor performance. Clustering of the B2 subgroups and VAGs and analysis of the assembly statistics were performed using a hierarchical clustering method in R (hclust). Abbreviations of the VAGs tested: genes coding for toxins—*hlyA* (α-hemolysin), *sat* (secreted autotransporter toxin, a serine protease), *cnf1* (cytotoxic necrotizing factor 1), *cdtB* (cytolethal distending toxin), and *tsh* (temperature-sensitive hemagglutinin); siderophores—*iroN* (salmochelin receptor), *iutA* (aerobactin synthesis, receptor), *ireA* (iron-regulated element, catecholate siderophore), and *fyuA* (yersiniabactin receptor); genes coding for adhesins—fimbria type P (*pap*GI, *pap*GII, *pap*GIII, *pap*A, *pap*C, and *pap*EF), *sfa/focDE* (type S fimbriae, *sfa*/*foc* S and F1C fimbriae), *afa/draBC*, adhesin *afa*/*dra* Dr antigen-binding adhesins (AFA I-III, Dr, F1845), *bmaE* (blood group M-specific adhesin), and *iha* (iron-regulated-gene-homologue adhesin); protectins—*kpsMT* II (group II capsule synthesis, e.g., K1, K5, K12), *kpsMT* III group III capsule synthesis (e.g., K3, K10, K54), and *traT* (surface exclusion; serum resistance associated); genes coding for invasins—*ibe*A-C (invasion of brain endothelium IbeA); genes coding for other genes—*cvaC* (microcin/colicin V; on plasmids with *traT*, *iss*, and *iuc/iut*), *ompT* (outer membrane protein T), *usp* (uropathogenic-specific protein, bacteriocin), PAI (*malX*, a PAI CFT073 marker), and *iss* (increased serum survival, outer membrane protein). Download FIG S5, PDF file, 0.2 MB. Copyright © 2021 Rodríguez et al. 2021 Rodríguez et al. https://creativecommons.org/licenses/by/4.0/ This content is distributed under the terms of the Creative Commons Attribution 4.0 International license.

10.1128/msphere.00868-21.7 FIG S6 Heatmap of the relative abundance of each VAG in each VAG group. Variables showing goodness-of-fit. Abbreviations of the VAGs tested: genes coding for toxins—*hlyA* (α-hemolysin), *sat* (secreted autotransporter toxin, a serine protease), *cnf1* (cytotoxic necrotizing factor 1), *cdtB* (cytolethal distending toxin), and *tsh* (temperature-sensitive hemagglutinin); siderophores—*iroN* (salmochelin receptor), *iutA* (aerobactin synthesis, receptor), *ireA* (iron-regulated element, catecholate siderophore), and *fyuA* (yersiniabactin receptor); genes coding for adhesins—fimbria type P (*pap*GI, *pap*GII, *pap*GIII, *pap*A, *pap*C, and *pap*EF), *sfa/focDE* (type S fimbriae, *sfa*/*foc* S and F1C fimbriae), *afa/draBC*, adhesin *afa*/*dra* Dr antigen-binding adhesins (AFA I-III, Dr, F1845), *bmaE* (blood group M-specific adhesin), and *iha* (iron-regulated-gene-homologue adhesin); protectins—*kpsMT* II (group II capsule synthesis, e.g., K1, K5, K12), *kpsMT* III group III capsule synthesis (e.g., K3, K10, K54), and *traT* (surface exclusion; serum resistance associated); genes coding for invasins—*ibe*A-C (invasion of brain endothelium IbeA); genes coding for other genes—*cvaC* (microcin/colicin V; on plasmids with *traT*, *iss*, and *iuc/iut*), *ompT* (outer membrane protein T), *usp* (uropathogenic-specific protein, bacteriocin), PAI (*malX*, a PAI CFT073 marker), and *iss* (increased serum survival, outer membrane protein). Download FIG S6, PDF file, 0.2 MB. Copyright © 2021 Rodríguez et al. 2021 Rodríguez et al. https://creativecommons.org/licenses/by/4.0/ This content is distributed under the terms of the Creative Commons Attribution 4.0 International license.

### Clonal relationships.

**FIG 6:**
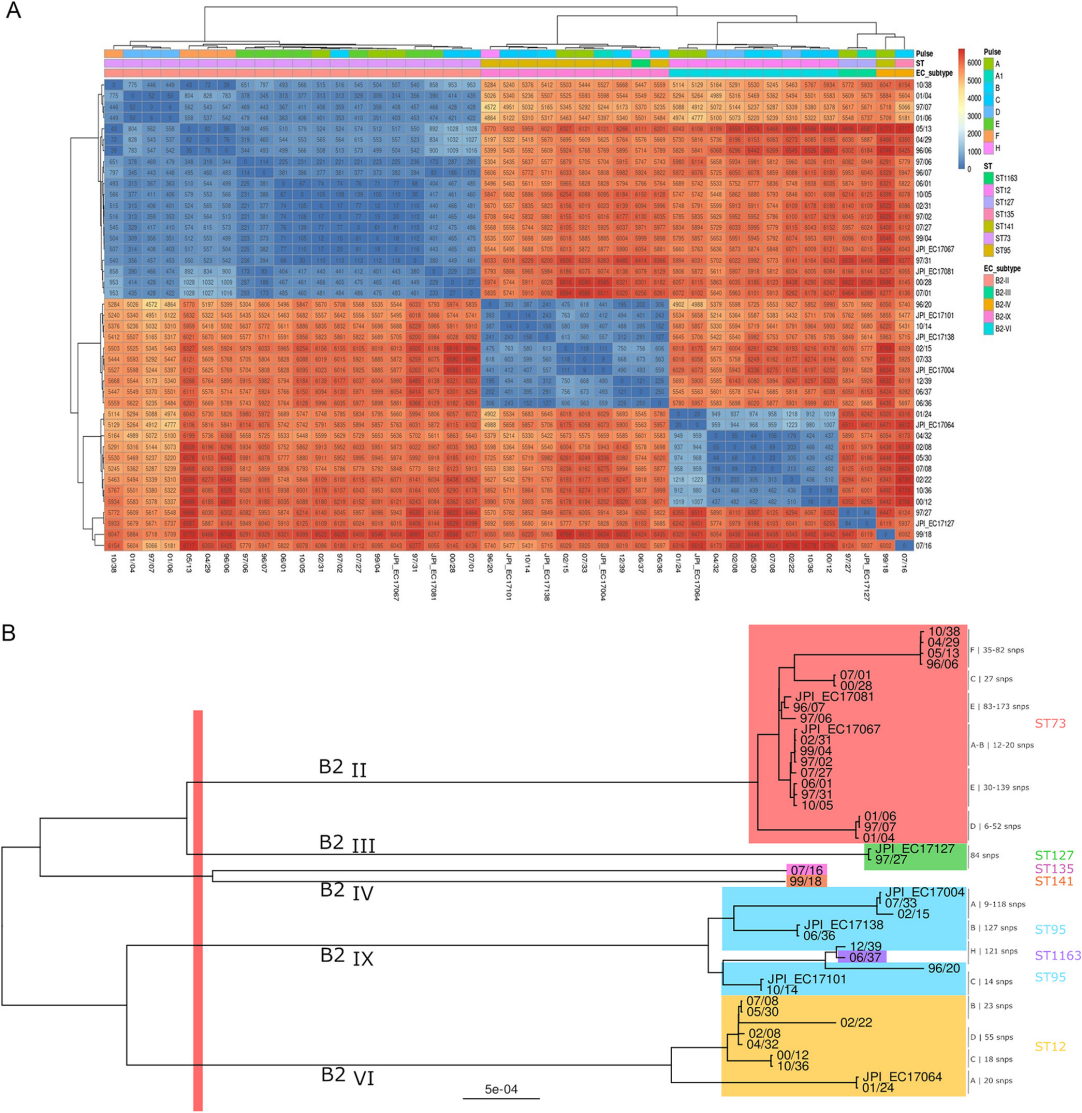
Characterization of B2 E. coli strains fully sequenced. (A) Heatmap of pairwise SNP distances based on the absolute differences of SNPs. The colors of the boxes identify the different SNP clades (B2-II-STc73, B2-IX-STc95, B2-IV-STc12, B2-III-STc127). (B) Maximum-likelihood phylogenetic tree of the whole genome created using FastTree (52). Clades are colored according to the ST in each B2 subgroup: B2-II ST73 (red), B2-IX-ST95 (blue), B2-IX-ST1163 (violet), B2-III-ST127 (green), B2-VI-ST12 (yellow), B2-IV-ST135 (pink), and B2-IV-ST141 (orange). SNP numbers represent the range of individual comparisons. Capital letters represent the PFGE type of these isolates (Table S1).

PFGE typing of isolates of B2 subgroups II to X revealed highly similar/identical patterns of XbaI-digested genomic DNA for isolates within B2 subgroups II (STc73), IX (STc95), VI (STc12), and III (STc127) collected during variable periods of time (2 to 17 years) (Table S1). Analysis of pairwise single nucleotide polymorphism (SNP) distances between isolates and the tree topology suggests the presence of different lineages of different origin for each ST, which showed identical or highly similar XbaI-digested genomic DNA patterns. It is of note that such lineages/PFGE types differ in a variable number of SNPs (6 to 173 SNPs) (Fig. 6A and B). Although genetic distances estimated as SNPs constitute the initial basis to determine the similarity between isolates, available thresholds for establishing such similarity are mostly based on outbreak investigations and conventional mutation rates, and so the interpretation of SNP/allelic values to determine the similarity of isolates remains challenging. Nonetheless, the similarity of the PFGE profiles and the time lapse between isolates suggest the successful and long-lasting transmission of certain lineages in the community.

## DISCUSSION

This work, one of the very few long-term longitudinal studies—and the first in Spain—to analyze the dynamics of E. coli phylogroups involved in BSI for a long period of time at a single center, reveals intertwined waves of CA+HCA BSI and HA BSI episodes caused by strains of both B2 and non-B2 phylogroups for decades. This would have occurred until BSI episodes caused by B2 populations of E. coli overtook those of non-B2 and the B2-I-STc131 became transiently predominant and predated new waves of other major B2 subgroups. Such dynamics are similar to that observed in the United Kingdom (17), but our study further suggests that the burdens of CA BSI and HA BSI infections are closely related, and genomic and epidemiological data suggest differences between the evolutionary pathways of different E. coli phylogroups and B2 subgroups.

B2-I (STc131) was the only lineage that significantly increased during the period analyzed, and this transient increase was due to the expansion of both C1 and C2 subclades (mostly ESBL negative) and, probably, to CTX-M-27 in the last years, according to the emergence of this subclade in the community after the end of this study (22). Although the epidemiology of STc131 has extensively been documented (11), this work and that by Kallonen et al. (17) are the only ones that reveal the coexistence of all STc131 subclades which, after a transient amplification, are incorporated into the E. coli population structure. The other predominant B2 subgroups varied greatly through the period studied, with some peaks of B2-II (STc73), B2-IX (STc95), and B2-VI (STc12) clones and also others (e.g., B2-VII, B2-X, and non-I/II) which are poorly documented in the literature. The identification of isolates of each predominant B2 phylogroup/ST, mostly collected from patients at the hospital emergency wards through a wide range of years (2 to 17 years), showing identical or highly similar PFGE types, and differing only in tens of SNPs (6 to 173 SNPs), is of utmost relevance and suggests a reservoir of highly stable “epidemic” strains in the community that would have evolved within or through different individuals or groups of related individuals (26). Note that the interpretation of SNP/allelic values to determine the similarity of isolates is still challenging because the thresholds currently used are based on outbreak investigations and reported mutation rates under laboratory conditions and, thus, can be extrapolated only to isolates collected in short periods of time.

Analysis of the population structure of commensal E. coli by Massot et al. demonstrated a shift In Western countries from phylogroups A and D in the 1970s to B2 and F in the 2000s which could be influenced by the increasing use of antibiotics (9). However, the steady increase of E. coli resistant to Cip and 3GCs observed since the early 1990s occurs in non-B2 groups and B2-ST131 subclade C and contrasts with the antibiotic susceptibility to Cip, 3GC, and other drugs of most predominant STs of B2 non-ST131 isolates and ST131 subclades A and C0 (this study and reference 27). Besides antibiotic pressure, significant changes in health care delivery and in human population structure determinants (age, interconnectedness, diet) have recently been suggested to explain the amplification of particular strains (28–30), but none of these factors fully justifies the results. Cip^R^ has recently been considered an advantage for the fitness of high-risk clones of E. coli such as ST131 subclade C and also of other bacterial species (56, 57). Some Cip^R^ clones are predominant in the elderly, with a sustained pattern in hospital (or outpatient center) admissions and personal clinical history that facilitates the acquisition of other antibiotic resistances (58). An increasing cumulative “elderly microbiota reservoir” of antimicrobial-resistant subpopulations of various bacterial species would be congruent with a high occurrence of CA+HCA BSI episodes at admission, with the age of the patients in our sample, and with the identification of identical PFGE types in groups of unrelated patients. However, the detection of B2 isolates of other non-ST131 clade C groups in people of all ages but predominantly in the range of 15 to 45 years and also in other nonhuman hosts (27–31), with the isolates eventually being able to circulate between them (26, 28), would reinforce that they are part of the normal microbiota. Note than the notion of “pandemic” implies the extended spread of the clones in the community, and the increase in number and exposure to different hosts and environments necessarily tends toward an increase in genetic diversity (11). In fact, there is a notion that the “high-risk clones” emerge from the epidemicity of commensals which precedes the spread of multiresistant bacterial clones (28, 32).

The correlation between VAG profiles and B2 subgroups reflects an apparent structure of B2 populations in agreement with the ecological niche specialization theory, considering that the niche of a population is the result of the niches occupied by all its individuals (7, 33–37), so that the niche is evolving itself (38, 39). Although the concordance of specific VAGs with isolates of B2-II and B2-IX subgroups is congruent with their classical categorization as “uropathogenic” E. coli, thereby explaining their bimodal distribution in different age groups, most B2-I strains (STc131 clade C) exhibit a low number of classical E. coli VAGs (40, 41). To explain the expansion of the STc131 and other emerging lineages, such as STc648 (phylogroup F), studies that have applied mathematical models to a high number of genomes suggest that negative-frequency-dependent selection of previously rare populations might have favored their increase (14, 41) and will foster the expansion of some others in the future. However, these studies are fully focused on the microorganisms and do not allow us to associate the observed changes with the type of hosts located either in hospital or in community compartments.

We are aware that the size of the sample analyzed, despite the high number of carefully randomized isolates analyzed, represents only 10% of the total number of BSI in our institution during the studied period. However, the long period analyzed at a single institution helps identify relevant epidemiological conditions that illustrate the expansion or particular E. coli populations observed in our and other areas during recent years. Currently, the B2 E. coli strains are relevant units of BSI pathogenicity, which should correlate with their success in particular microecological landscapes, in part determined by recent interventions exerted on particularly fragile human populations (mostly elderly patients), and also because of the cumulative effect of interventions over a lifetime. This view is in agreement with the concept of incorporating ecological features into the identification of the fundamental units of bacterial diversity (30) and pathogenicity. In fact, particular clones and lineages are differentially represented in a “human microbiota reservoir” flowing from the community to the hospital and vice versa, where they can either be selected or coexist as predicted by an evolutionarily stable strategy (30). The evolutionary trajectories of recently amplified major lineages indicate the relevance of undetected selective events, which could be further amplified by the acquisition of antimicrobial resistance in settings under (or not) antibiotic pressure (29, 30, 42, 53). The early detection of the abundance and diversity of B2 subgroups of clinical significance for BSI in metagenomic samples of community-based individuals and the analysis of common causes that enhance their selection, either in the hospital, in nursing homes, or in the community, are priority research challenges that warrant attention in an era that favors pandemics of microorganisms and antimicrobial resistance (43).

## MATERIALS AND METHODS

### Study design.

Ramón y Cajal University Hospital is a tertiary-level public health center with 1,155 beds that provides attention to 600,000 inhabitants in the northern area of Madrid (Spain), which reflects a pyramid-age of “declining type,” has full access to primary care, and has a predominantly medium-high socioeconomic level. Of the total 21,695 positive blood cultures detected between January 1996 and December 2016, we identified 7,165 E. coli isolates that represented 1 isolate per patient and per BSI episode. A sample of nearly 10% of this E. coli collection, stratified by sex, age, and antimicrobial resistance pattern, was sorted by statistical randomization (Stata Statistical Software, release 17; StataCorp LLC, College Station, TX) and was further analyzed (649 E. coli isolates from 339 females and 310 males; <1 to 98 years of age). The study was approved by the Ethics Committee of our hospital.

BSI are classified as hospital acquired (HA), community acquired (CA), and community-onset health care associated (HCA), according to the date of the sample collection after patient admission and the patient exposure to hospitals before the BSI episode (19, 42). Due to the inaccessibility of all the medical records of patients enrolled in this study, and their advanced age, we classified the episodes into HA (if the blood culture was obtained in the intensive care unit [ICU] or surgical or medical areas after 48 h of admission) and CA+HCA (if the blood culture was obtained in the hospital emergency wards or at the outpatient centers) categories. UTIs were considered the origin of BSI if E. coli was recovered from both the urine and blood samples, with a difference of ±24 h.

### Characterization of the bacterial isolates.

Blood culture isolates of E. coli are routinely frozen and stocked in skimmed milk at −70°C and were subcultured onto brain heart infusion agar prior to analysis. Bacterial susceptibility to 13 antibiotics (ampicillin, amoxicillin-clavulanic acid, cefotaxime, ceftazidime, meropenem, nalidixic acid, ciprofloxacin, streptomycin, kanamycin, gentamicin, tetracycline, chloramphenicol, and trimethoprim-sulfamethoxazole) was analyzed by the disk diffusion method (44).

Multiplex PCR assays allowed classifying the E. coli isolates into major phylogenetic groups A, B1, B2, C, D, E, and F (45); B2 subgroups (B2-I to -X) (46); and B2-I-STc131 E. coli serotypes (O16/O25b), clades (H41, H22, and H30), and the H30 subclades C0 (H30, fluoroquinolone susceptible [FQ^S^]), C1 (H30-R, fluoroquinolone resistant [FQ^R^]), C2 (H30-Rx, FQ^R^ + *bla*_CTX-M-15_), and C1-M27 (H30-Rx, FQ^R^ + *bla*_CTX-M-27_) (46, 47). STc131 isolates were further analyzed by pulsed-field gel electrophoresis (PFGE). The presence of 29 virulence-associated genes (VAGs) was determined for all B2 isolates by PCR (6, 48).

A clonal relationship between B2 isolates was preliminary established by pulsed-field gel electrophoresis (PFGE) according to the PulseNet website (https://pulsenetinternational.org/protocols/pfge/). Isolates showing the same or highly related PFGE profiles (1 to 3 bands) were selected for WGS (Table S1).

### Whole-genome sequencing.

Forty-five isolates corresponding to isolates showing similar or highly related PFGE types were selected for WGS. DNA was extracted from 5 ml of overnight cultures using the Wizard genomic DNA purification kit (Promega Corp., Madison, WI, USA), and DNA concentration was measured using a Qubit fluorometer and Nanodrop 2000 (Thermo Scientific, Waltham, MA, USA). WGS was performed using the Illumina NovaSeq 6000 platform (Oxford Genome Center, Wellcome Centre for Human Genetics, Oxford, United Kingdom) with 2% 150-bp paired-end reads. Quality control and sequence filtering were done using the FastQC v.0.11.8 (https://www.bioinformatics.babraham.ac.uk/projects/fastqc/) and Prinseq-lite-0.20.3 (http://prinseq.sourceforge.net/) tools, respectively.

The paired-end reads were *de novo* assembled using SPAdes v.3.14.1 (48), and then they were annotated with Prokka (49) The phylogenetic analysis was obtained using PATO (50) and fasttree2.1 (51). The heatmap resulted from a whole-genome SNP pairwise comparison (PATO), and the tree was made with ggtree (52). *In silico* multilocus sequence type (MLST) assignment was performed using MLST v2.16.1 (https://github.com/tseemann/mlst).

### Statistical analysis.

To calculate statistical significance, the chi-square test, a 2-sample *t* test for normally distributed variables, and Kendall’s correlation were used, considering *P* values of <0.05 to be statistically significant.

### Data availability.

The sequences of the genomes have been registered in the BioProject database with the reference PRJNA775650. The references for the BioSample accessions are SAMN22610064 to SAMN22610106.

10.1128/msphere.00868-21.8 FIG S7 Heatmap of 29 VAGs (presence/absence). The heatmap was annotated with the FimH allele, ST131 serotype, patient age, and sex. Abbreviations of the VAGs tested: genes coding for toxins—*hlyA* (α-hemolysin), *sat* (secreted autotransporter toxin, a serine protease), *cnf1* (cytotoxic necrotizing factor 1), *cdtB* (cytolethal distending toxin), and *tsh* (temperature-sensitive hemagglutinin); siderophores—*iroN* (salmochelin receptor), *iutA* (aerobactin synthesis, receptor), *ireA* (iron-regulated element, catecholate siderophore), and *fyuA* (yersiniabactin receptor); genes coding for adhesins—fimbria type P (*pap*GI, *pap*GII, *pap*GIII, *pap*A, *pap*C, and *pap*EF), *sfa/focDE* (type S fimbriae, *sfa*/*foc* S and F1C fimbriae), *afa/draBC*, adhesin *afa*/*dra* Dr antigen-binding adhesins (AFA I-III, Dr, F1845), *bmaE* (blood group M-specific adhesin), and *iha* (iron-regulated-gene-homologue adhesin); protectins—*kpsMT* II (group II capsule synthesis, e.g., K1, K5, K12), *kpsMT* III group III capsule synthesis (e.g., K3, K10, K54), and *traT* (surface exclusion; serum resistance associated); genes coding for invasins—*ibe*A-C (invasion of brain endothelium IbeA); genes coding for other genes—*cvaC* (microcin/colicin V; on plasmids with *traT*, *iss*, and *iuc/iut*), *ompT* (outer membrane protein T), *usp* (uropathogenic-specific protein, bacteriocin), PAI (*malX*, a PAI CFT073 marker), and *iss* (increased serum survival, outer membrane protein). Download FIG S7, PDF file, 0.6 MB. Copyright © 2021 Rodríguez et al. 2021 Rodríguez et al. https://creativecommons.org/licenses/by/4.0/ This content is distributed under the terms of the Creative Commons Attribution 4.0 International license.
