# Supplementary figures and images for: A 21-Year Survey of Escherichia coli from Bloodstream Infections (BSI) in a Tertiary Hospital Reveals How Community-Hospital Dynamics of B2 Phylogroup Clones Influence Local BSI Rates
Source: mSphere. 2021 Dec 22;6(6):e00868-21. doi: 10.1128/msphere.00868-21 (PMC8722714; doi:10.1128/msphere.00868-21)

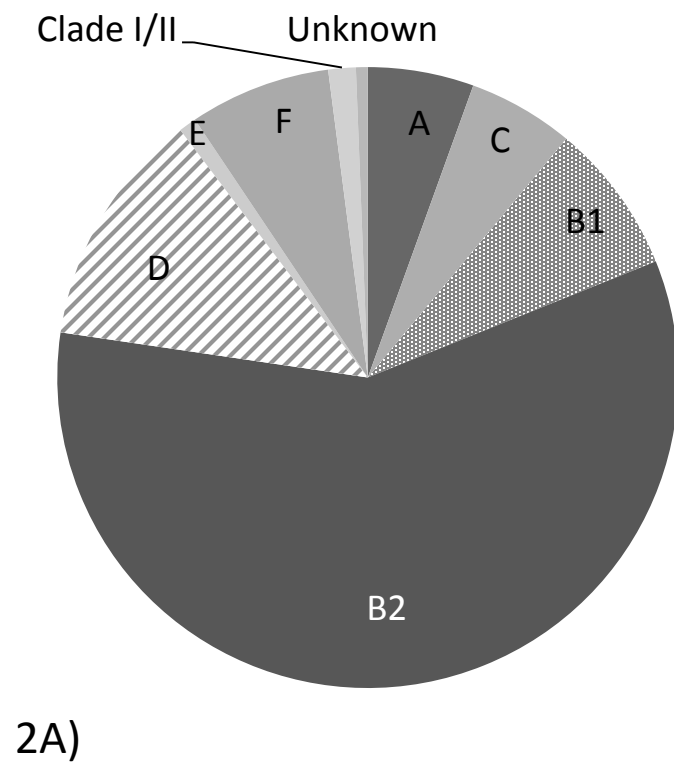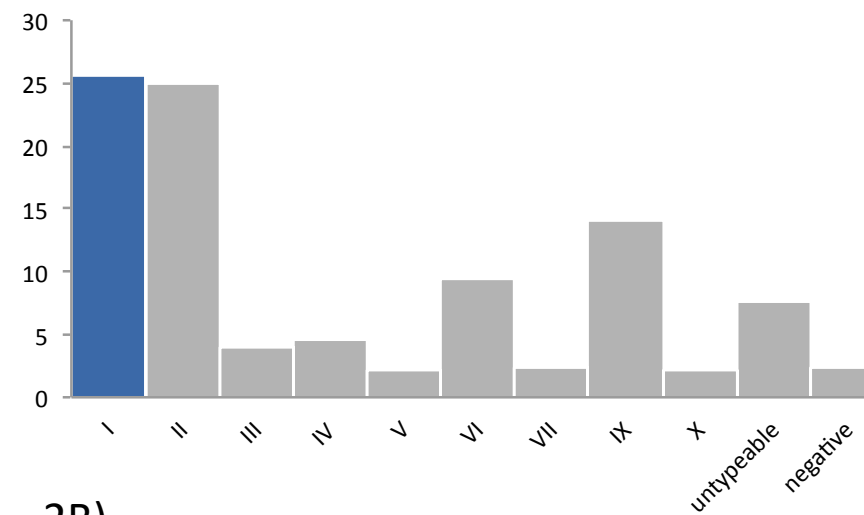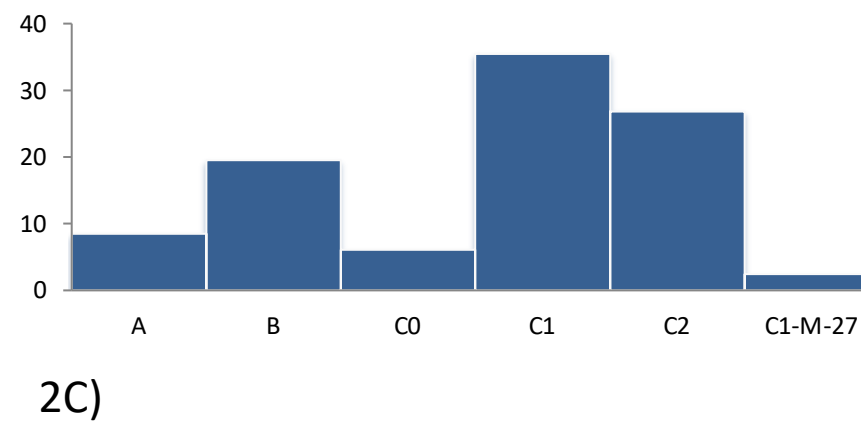

Figure S1

Supplement: FIG S1 [file msphere.00868-21-sf001.pdf]

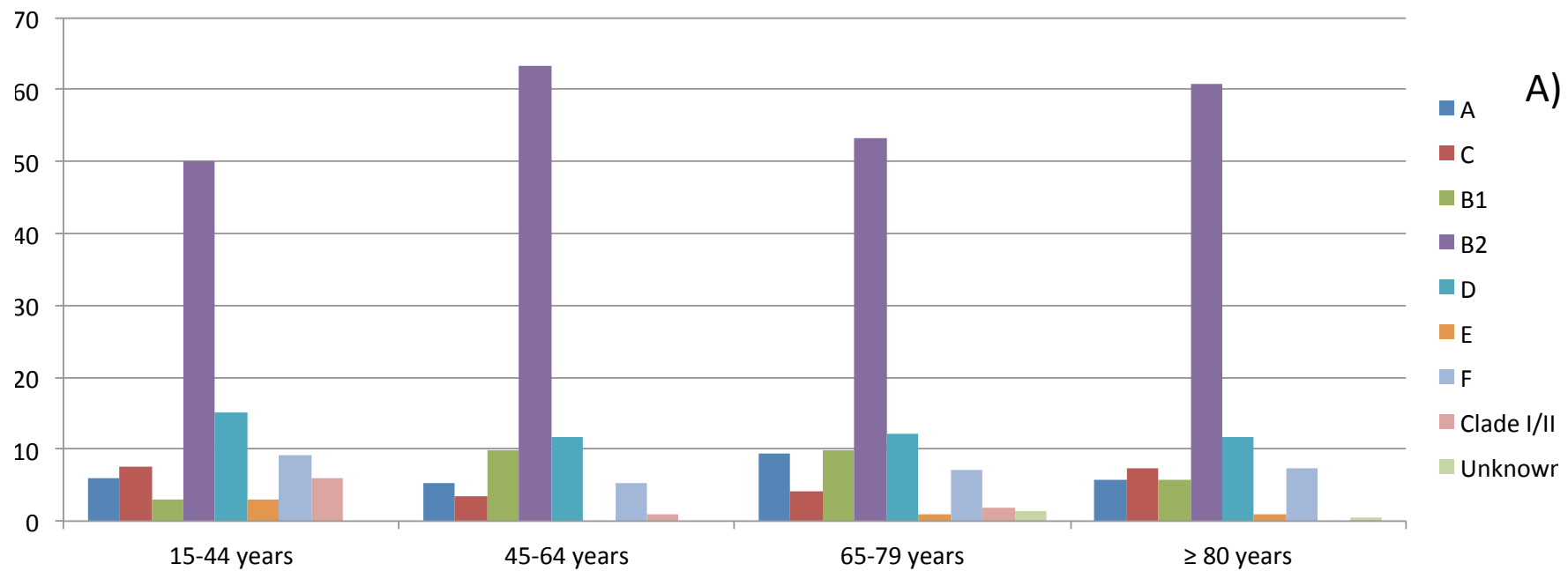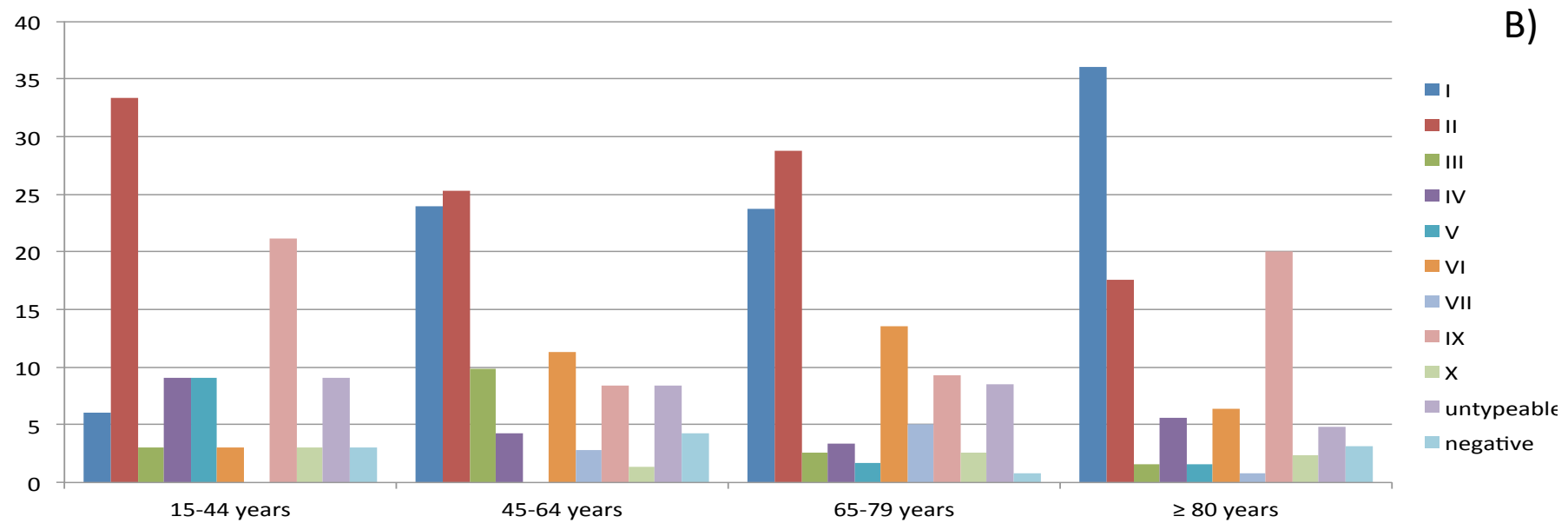

Figure S2

Supplement: FIG S2 [file msphere.00868-21-sf002.pdf]

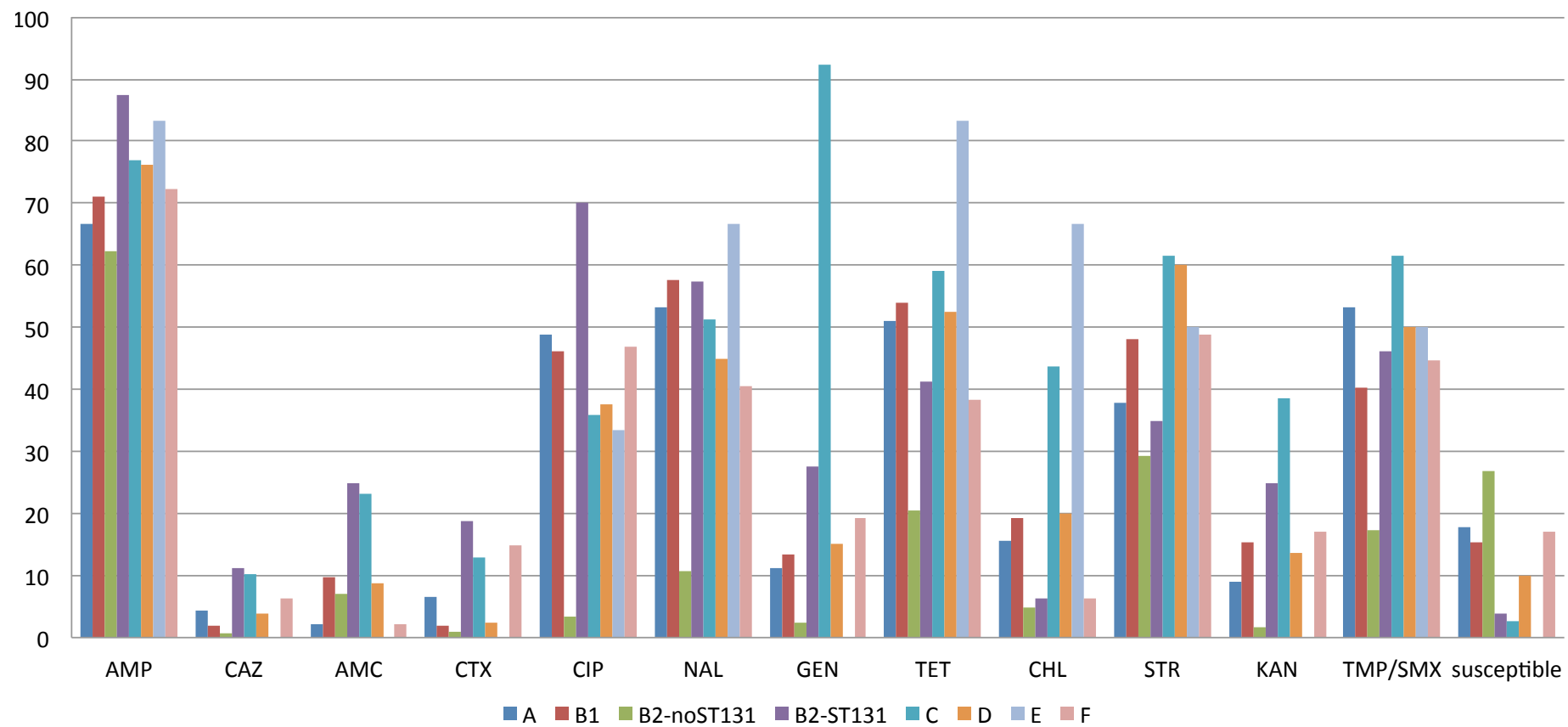

Figure S3

Supplement: FIG S3 [file msphere.00868-21-sf003.pdf]

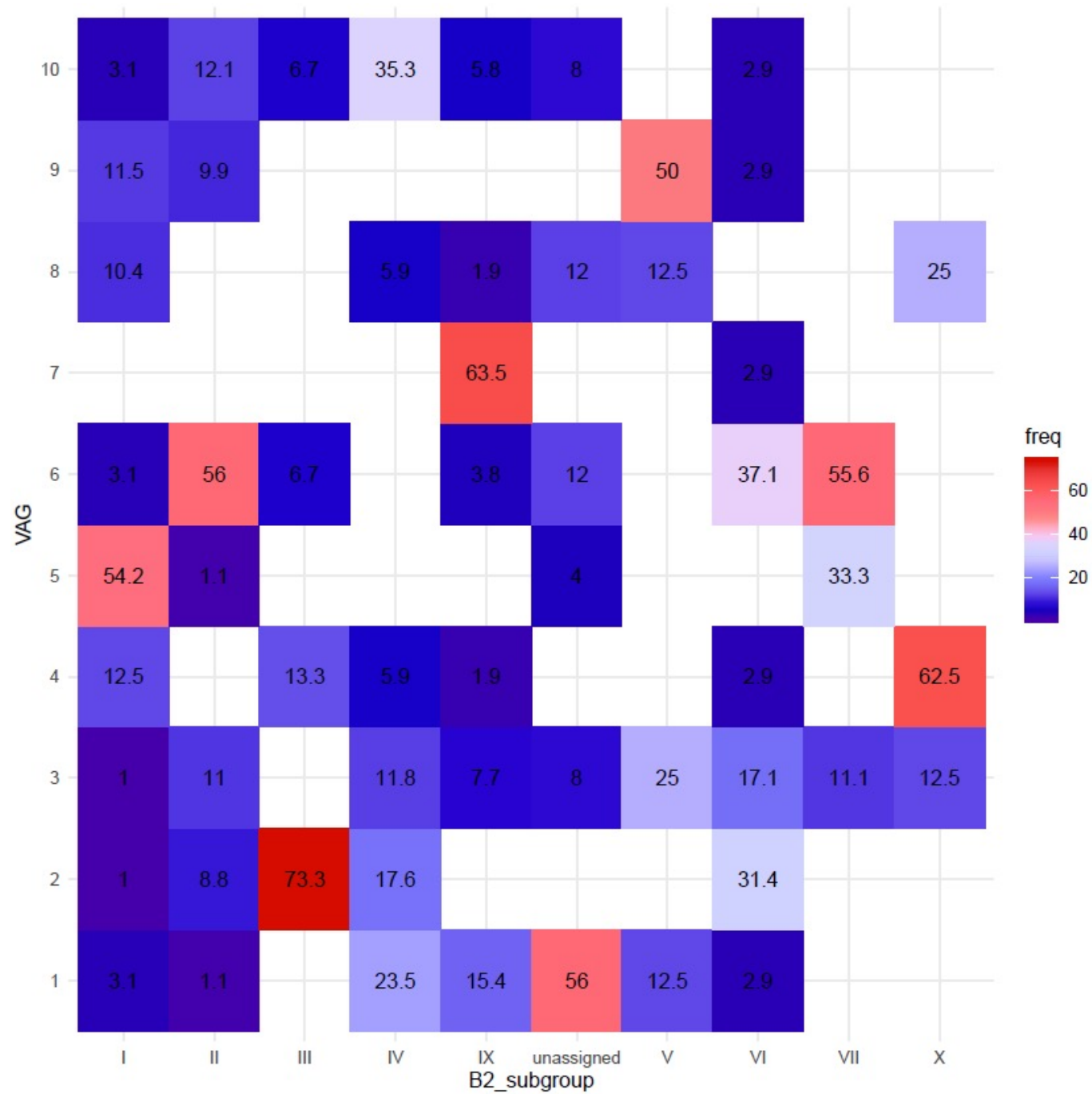

Figure S4

Supplement: FIG S4 [file msphere.00868-21-sf004.pdf]

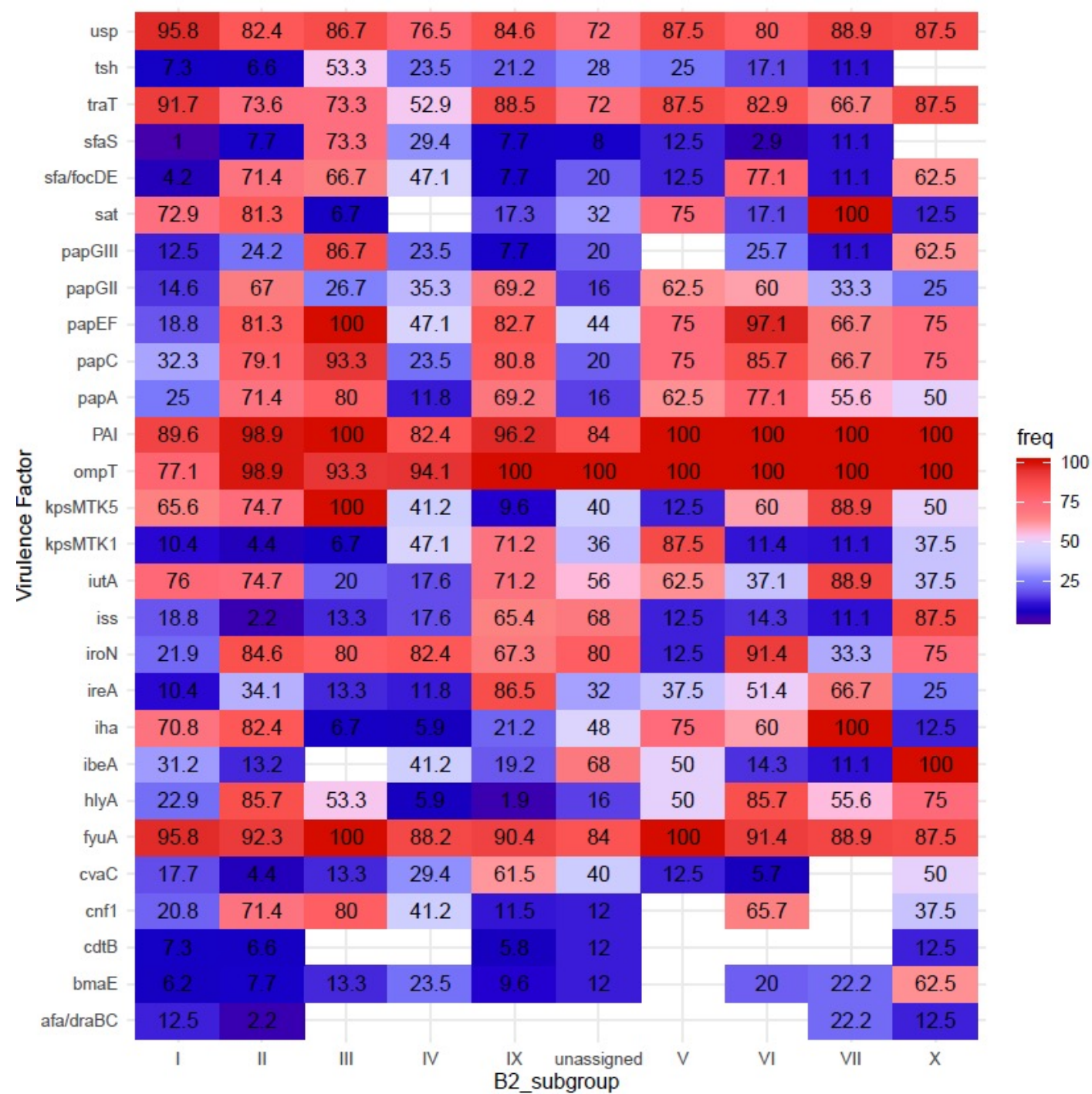

Figure S5

Supplement: FIG S5 [file msphere.00868-21-sf005.pdf]

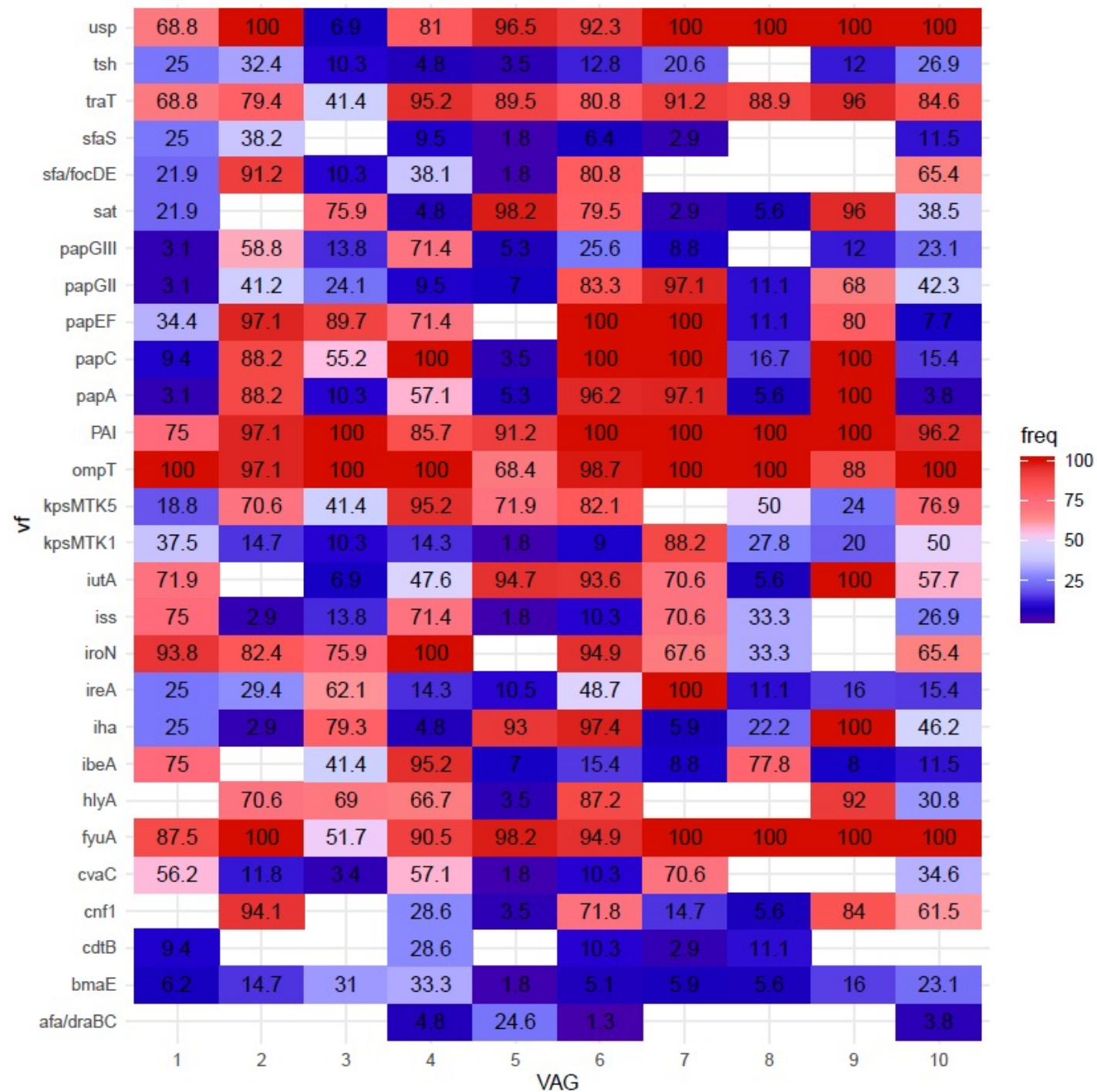

Figure S6

Supplement: FIG S6 [file msphere.00868-21-sf006.pdf]

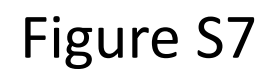

Supplement: FIG S7 [file msphere.00868-21-sf007.pdf]
