# Supplementary material for: A 21-Year Survey of Escherichia coli from Bloodstream Infections (BSI) in a Tertiary Hospital Reveals How Community-Hospital Dynamics of B2 Phylogroup Clones Influence Local BSI Rates
Source: mSphere. 2021 Dec 22;6(6):e00868-21. doi: 10.1128/msphere.00868-21 (PMC8722714; doi:10.1128/msphere.00868-21)
Supplement: TABLE S1 [file msphere.00868-21-st001.doc]

**Table S1. Relationship between major *Escherichia coli* clonal lineages causing BSI at HURyC (1996-2016)**

| **EC Subtype** | **PFGE type** | **Isolates**  **(sequenced/ total number)** | **ST** | **Year of isolation**  **(number of isolates)** | **Ward**  **(number of isolates in this ward)** |
| --- | --- | --- | --- | --- | --- |
| **B2-II** | A | 4/29 | ST73 | 1997(3), 1998(1), 1999(1), 2001(1), 2002(2), 2003(3), 2004(3), 2005(1), 2006(1), 2008(2), 2009(1), 2011(3), 2012(1), 2013(2), 2014(2),  2015(2) | Emergency (20), Emergency-ICU (1), Internal Medicine (3), Cardiology (1), Nephrology (1), Gastroenterology (1), unknown (2) |
|  | B | 2/4 | ST73 | 1997(1), 1998(1), 2001(1), 2004(1) | Emergency (3), Cardiology (1) |
|  | C | 2/3 | ST73 | 2000(1), 2002(1), 2007(1) | Internal Medicine (1), Gastroenterology (1), unknown (1) |
|  | D | 2/6 | ST73 | 1997(1), 2001(1), 2002(1), 2006(1), 2015(2) | Emergency ward (3), Emergency-Surgery (2), Internal Medicine (1) |
|  | E | 7/14 | ST73 | 1996(1), 1997(2), 2003(2), 2004(1), 2006(1), 2007(1), 2010(1), 2011(1), 2015(3), 2016(1) | Emergency (8), Emergency-Surgery (1), Neurology (1), General surgery (1), (3) |
|  | F | 3/4 | ST73 | 1996(1), 1999(1), 2005(1), 2010(1) | Emergency (2), Internal Medicine (1), Unknown(1) |
|  | H | 1/3 | ST73 | 2007(1), 2008(1), 2009(1) | Emergency (2), Urology(1) |
| **B2-III** | A, A1 | 2/10 | ST127 | 1999(1), 2004(1), 2005(1), 2006(1), 2007(1), 2010(1), 2014(1), 2015(1), 2016(2) | Emergency (5), Internal Medicine (1), Hematology (1), Unknown (3) |
| **B2-IV** | A | 1/5 | ST141 | 1999(3), 2008(1), 2009(1) | Emergency (3), Emergency-surgery(2) |
|  | B | 1/1 | ST135 | 2007(1) | Emergency-surgery (1) |
| **B2-VI** | A | 2/8 | ST12 | 2001(1), 2002(2), 2003(2), 2009(1), 20014(1), 2015(1) | Emergency (4), Urology (1), Hepatology transplant unit (1), Neurology (1), Nephrology transplant unit (1) |
|  | B | 2/3 | ST12 | 2005(1), 2007(2) | Emergency (1), Traumatology (1), Infectious Diseases (1) |
|  | C | 2/4 | ST12 | 2000(1), 2008(1), 2010(1), 2012(1) | Emergency-surgery (1), Surgery (1), ICU-Neurosurgery  (1), Short Stay Unit (1) |
|  | D | 3/11 | ST12 | 2002(2), 2004(1), 2012(1), 2014(3), 2015(2), 2016(2) | Emergency (7), Urology (1), Internal Medicine (1), Infectious Diseases (1), Neurosurgery (1) |
| **B2-IX** | A, A1 | 2/7 | ST95 | 2007(2), 2008(1), 2011(3), 2013(1), 2016(1) | Emergency(4), Internal Medicine (3), Oncology(1) |
|  | B | 4/10 | ST95 | 1999(1), 2002(1), 2006(1), 2008(1), 2010(1), 2012(2), 2014(1), 2015(1), 2016(1) | Emergency (6), Emergency-surgery (1), Gastroenterology (1), Infectious Diseases (1), Internal Medicine (1) |
|  | C | 2/3 | ST95 | 2010(1), 2011(1), 2016(1) | Emergency (1), Internal Medicine (1), Unknown (1) |
|  | G | 1/2 | ST95 | 2002(2) | Emergency (1), Endocrinology (1) |
|  | H | 3/6 | ST95,  ST79, ST1163 | 1996(1), 1998(2), 2006(2), 2008(1) | Emergency (2), Emergency surgery (2), Internal Medicine (1), Traumatology (1) |

Abrreviattions. EC= *Escherichia coli;* PFGE = pulsed field electrophoresis; ST=Sequence type
